# Why Acute Undernutrition? A Qualitative Exploration of Food Preferences, Perceptions and Factors Underlying Diet in Adolescent Girls in Rural Communities in Nigeria

**DOI:** 10.3390/nu16020204

**Published:** 2024-01-08

**Authors:** Mercy E. Sosanya, Jeanne H. Freeland-Graves, Ayodele O. Gbemileke, Oluwatosin D. Adesanya, Oluwaseun O. Akinyemi, Samuel O. Ojezele, Folake O. Samuel

**Affiliations:** 1 Department of Nutritional Sciences, University of Texas at Austin, Austin, TX 78712, USA; 2 Department of Nutrition and Dietetics, The Federal Polytechnic, Bauchi 740102, Nigeria; otochidorothy@fptb.edu.ng; 3 Nutrition International, Abuja 900108, Nigeria; 4 Department of Health Policy and Management, College of Medicine, University of Ibadan, Ibadan 200285, Nigeria; 5 Department of Human Nutrition and Dietetics, College of Medicine, University of Ibadan, Ibadan 200132, Nigeria

**Keywords:** adolescent girls, free lists, focus group discussions, food preferences, perception of nutritional value, malnutrition, nutrition knowledge, inequitable food access, courtship and marriage practices, Bauchi, Nigeria, rural areas

## Abstract

Background: Adolescent girls are nutritionally vulnerable due to their rapid growth and increased nutrient requirements. Nigeria has the sixth-largest population in the world. This study qualitatively explored the food preferences, perceptions of nutritive value and factors underlying food consumption of adolescent girls in rural communities in Nigeria. Methods: The data were collected via the free listing of foods and focus group sessions conducted in the Hausa language with 48 unmarried adolescent girls. The discussions were audio-recorded, transcribed, translated into English, and analyzed using a deductive thematic framework. Results: The mean age of the respondents was 13.0 ± 2.7, and almost half (48%) had a primary school education. A total of 19 and 23 foods were identified as preferred, and perceived as nourishing, respectively. The top 10 foods present on both free lists overlapped considerably in terms of cognitive salience. The focus group themes included nutrition knowledge, food preferences, autonomy, household food allocation, courtship practices, and agricultural landscapes and economic access. The participants had minimal knowledge of nutrients and food groups, and their preferred foods were limited in diversity. The key factors in food preferences were desirable health effects, sensory attributes, and the contribution of foods to a desirable body image for marriage. Household food choices depended on parents. Thus, a desire for independence was an incentive for early marriage, mostly at 13 to 17 years. Gender inequities in household food distribution (quantity) and animal protein intake were reported. The participants believed that boys need more food for strength to impregnate girls. As part of a courtship practice, the girls received gifts of animal source foods from potential suitors. The food options were limited by financial challenges and low agricultural diversity. Conclusion: To interrupt the cycle of inadequate food consumption and undernutrition in these adolescent girls, policy makers need to promote nutrition education and address the underlying determinants of inequitable access to nutritious foods.

## 1. Introduction

Adolescence is the stage of life between childhood and adulthood, from the ages of 10 to 19 years [1]. There are 1.2 billion adolescents, comprising approximately 16% of the global population [2]. This age group is nutritionally vulnerable due to increased nutrient needs, food insecurity, eating patterns, changing lifestyles, and susceptibility to environmental influences [3]. Adolescent girls are particularly prone to experiencing undernutrition, including anemia, because of iron losses in menstruation and rapid growth, to an extent that surpasses that in every other phase of life (with the exception of the first year) [4]. Girls who become pregnant during adolescence are at elevated risk of complications, since they may still be growing [4]. The enhancement of nutrition in this phase of life is critical to the improvement of the nutritional wellbeing of the entire population.

Globally, Nigeria has the sixth-largest population, estimated at 224.3 million, with 63% of the population experiencing multidimensional poverty [5,6]. This research was conducted in Bauchi, a predominantly rural state located in the northeastern part of Nigeria [7]. This area has an exceptionally high prevalence of multidimensional poverty (73.9%) [5]. Among the 36 states in Nigeria, Bauchi State has the second-highest incidence of acute malnutrition among adolescent girls and women 15–49 years (13% with mid-upper-arm circumference ≤221 mm) [8]. In adolescent girls (15 to 19 years), the nationwide prevalence of acute malnutrition was found to be over four times greater than that of adult women (20 to 49 years), at 19% vs. 4%, respectively. By extrapolation, the acute malnutrition rates for adolescent girls in the Bauchi state would even be higher than the 13% for all women of reproductive age [8]. This high prevalence underscores the urgency of exploring factors that underlie the food consumption of these adolescent girls, as a basis for developing effective interventions to improve nutrition and health outcomes in this population.

Bauchi State has 55 ethnic groups, of which the Hausa/Fulani are the most dominant [9]. It has a large Muslim majority in the northern communities, with a more heterogenous southern area that includes a Christian minority [10]. Polygamy and large household sizes are common, with Bauchi state having the highest fertility rate (7.2) in the northeast, and second highest in the country [11]. Evidence exists that food is produced in sufficient amounts in Bauchi State, as an estimated 80% of the inhabitants of the state are involved in subsistence agriculture [12]. Foods produced locally include crops such as rice, guinea corn, maize, millet, legumes, sweet potatoes, and watermelons, as well as poultry and ruminant animals [13]. However, poverty limits the ability of numerous households to reliably obtain food in adequate quantities and quality [14]. It has been documented that the Hausa culture is patrilineal, with men occupying preeminent economic and social positions, since they are culturally responsible for earning income and providing for their families [15,16]. Thus, the food consumed by households is often purchased by male household heads, as Islam, the predominant religion, dictates that married women of reproductive ages should remain confined to the family compound [16]. The economic status and social mobility of men empowers them to consume high-quality, luxury foods (including meat) in social men’s groups that are usually not available for household members, including adolescent girls [15,16]. Thus, environmental and social factors that underscore food access may influence the food choices of adolescent girls in Bauchi State.

Numerous factors may influence the diets of adolescent girls, including knowledge of food and nutrition, perceptions of healthful foods, and taste preferences [17]. Poor knowledge of nutrient requirements and food groups has been associated with undernutrition among adolescent girls in Bangladesh [18]. In Indonesia, one-point increments in nutrition knowledge in adolescent girls were associated with an increase in height-for-age with a standard deviation of 0.037 [19]. In Hong Kong, a recent study identified barriers to healthy eating among adolescents, including a lack of knowledge concerning recommended amounts and the long-term health effects of specific food types [20]. In a Ghanaian adolescent population with limited baseline knowledge concerning iron, a recent nutrition education intervention increased knowledge of iron-rich foods and, ultimately, iron intake [21].

Dietary habits and food selection may be influenced by perceptions of healthful food and taste preferences [22,23]. Food preferences are quantitatively related to food consumption, and may significantly influence nutrient intake [24]. For example, the consumption of healthy snack foods was higher among adolescent girls in Europe who perceived that current food intake would influence their future health compared to girls who did not [25]. Baxter et al. (2000) have shown that older children consume approximately 0.92 times the serving size of their preferred foods, 0.54 times for food to which they are indifferent, and 0.11 times for foods they dislike [24]. In studies in low- and middle-income countries, adolescents exhibited a personal dislike for dairy products, green leafy vegetables, animal flesh, and fruits, and a preference for sugary, salty, and fatty foods [26]. Thus, evidence exists that food preference is an underlying factor in food choices and the intake of critical nutrients, and that it is often dependent on sensory properties, including food color, shape, taste, and texture [27,28]. Associations between area of residence (rural/urban) and eating habits and food preferences were documented in a study on elementary school students in South Korea [29]. Additionally, the food choices and nutritional status of adolescent girls in Bangladesh were reportedly affected negatively by early marriage and gender inequity in intra-household food allocation, as the needs of male siblings, in-laws, and husbands are often prioritized [23].

Several quantitative studies have attempted to characterize the dietary pattern and nutritional conditions of adolescent girls in Nigeria [30,31,32]. The prevalence of underweight among girls in these studies ranged widely, from 11.1–46.8% [30,31,32]. The characteristic diets were high in refined cereals, and low in dietary diversity and animal protein consumption [30,31,32]. Nonetheless, there is limited information on attributes that may affect food intake in this population. Thus, this study employed a qualitative design to explore the food preferences, perceptions, and factors affecting dietary consumption among adolescent girls in selected rural communities in Bauchi State, Nigeria.

## 2. Methods

### 2.1. Theoretical Framework

**Figure 1:**
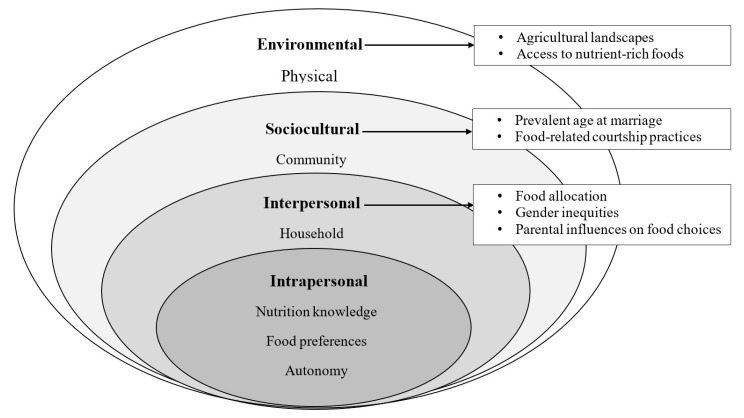
Ecological model of factors influencing food choices among adolescent girls in Bauchi State, Nigeria. Adapted with permission from Ref. [36]. 2008, Glanz K, Rimer BK, Viswanath K. Health Behavior and Health Education: Theory, Research, And Practice: John Wiley & Sons.

The ecological model of health behavior propounded by McLeroy et al. (1988) was adapted as the framework for this study (Figure 1) [33]. This theory focuses on the interactions between individuals and their physical and sociocultural environments. The model emphasizes the interplay between the intrapersonal characteristics and skills of the individual (adolescent girl) and proximal interpersonal (household) influences, as well as broader determinants of food consumption behavior, including sociocultural (community) and environmental (resource-related) factors. Adolescent girls in this study were at the core of their ecological context, and their lived experiences in relation to food consumption were discussed [34]. The intrapersonal characteristics of these adolescent girls influence the types of foods they consume. The other ecological levels (household, community, and environment) can either strengthen or weaken the ability of these girls to choose optimal diets [35].

### 2.2. Study Design, Area and Sites

Qualitative data were collected as part of a broader study in target areas [37] of the Oxfam LINE Project. The larger study documented the food consumption of 718 vulnerable farming households in rural areas of six Local Government Areas (LGAs) in Bauch State: Gamawa and Shira in the northern senatorial district, Ningi and Darazo in the central district, and Alkaleri and Tafawa Balewa in the south. The Bauchi State covers two different agroecological zones—the mountainous Sudan savannah, a semi-arid area in the south, and the Sahel savannah, in the north, composed mainly of thorny shrubs [38].

### 2.3. Study Population

In each of the six LGAs, three wards were selected by balloting, and two–three unmarried adolescent girls (10–19 years) were purposively chosen from the sampled households in each ward, for a sum of eight girls per LGA. One free listing and focus group session was held with eight adolescent girls in each of the six LGAs, for a total of 48 participants. All participants and their parents provided informed consent. Ethical approval was obtained from the Bauchi State Health Research Ethics Committee (BSMOH/NREC/12/05/2013/2017/22) (approved on 19 June 2017). Each audio-recorded discussion was conducted in the Hausa language by a facilitator, assisted by a scribe who compiled notes throughout the discussion.

## 3. Data Management

### 3.1. Data-Collection Instrument

The free listing and focus group interviews followed procedures in the Focus Group Discussion Guide, which was developed by the research team. Before each session, the female facilitator and note taker introduced themselves and started an ice-breaking session. The facilitator explained the aims of the study and the confidentiality of the outcomes. The food preferences and perceptions of nourishing foods described by the study participants were explored via free listing, an established anthropological and ethnographic technique used to generate ordered lists of domain elements as they occur to the minds of interviewees [39,40,41]. This technique investigates how individuals in groups conceptualize lists of items that generally belong together in one cognitive domain [42]. Free listing is based on the cognitive mapping theory, which posits that individuals have mental models that represent their perceptions, knowledge, and beliefs about a topic [43]. These mental models can be characterized by exploring how participants organize words and/or images of the components associated with a topic, in order of importance. The item importance is derived by calculating the Smith’s salience index [44,45,46]. The computation of Smith’s salience indices is one of the methods employed in cultural-domain analysis to summarize and contrast cognitive data [43]. The determination of the Smith’s salience index from free listing has been utilized in a variety of studies in areas such as cardiac-failure management [41], antenatal care [47], education [48], alcohol-use investigations [49], adolescent depression [50], psoriasis therapy [51], and many others.

Three basic assumptions underlie free listing: (a) the order in which people free list is based on familiarity with the subject; (b) individuals who are more familiar with the subject list more terms than those who are less familiar; and (c) the most frequently mentioned terms coincide with locally prominent elements [52]. In each focus group, the participants generated lists of foods that they preferred and perceived as nourishing, in response to the following prompts: (1) “Can you list the foods that you commonly prefer to eat?” and (2) “Can you list some foods that you consider to be nourishing?” The facilitators encouraged the participants to list as many foods or dishes as possible, without any limitations, and these were recorded according to the order in which they were mentioned. Additional semi-structured and open-ended questions were asked concerning the factors influencing food consumption, with each focus group lasting approximately 60 min.

### 3.2. Data Analyses

The audio-recorded data from the focus group interviews were transcribed and translated from Hausa into English. The foods or dishes mentioned in the free lists generated from the six LGAS were placed in Excel, according to the order in which they were mentioned in each LGA. Foods or dishes listed in singular and plural form were unified as one. For instance, “egg” or “eggs” were named in one column as “egg.” Additionally, different foods or dishes from the same food category were unified; for instance, “spaghetti,” “macaroni,” or “couscous” were renamed “pasta.” Most composite Nigerian dishes derive their names from the main ingredients used in their preparation [53,54,55]. Composite dishes with more than one main ingredient were separated into the main food components, and these components were ranked according to the order in which they were mentioned. For example, in the dish “beans and yam porridge,” “beans” was ranked first, while “yam” was placed next on the list and ranked immediately below “beans.” Finally, multiple responses about a specific food or dish were considered only the first time these were mentioned, respecting their position on the list of each focus group [56].

The free lists were entered into Free List Analysis in the R environment using Shiny (FLARES) software version 1.0, designed for cultural analyses of free lists [57]. To determine the most cognitively relevant foods with respect to preference and perceptions of nourishment value, a salience index (Smith’s *S*) was generated from the formula *S* = ((*L − R_y_* + 1)/*L*)/*N*, where *L* = the length of each individual list, *R_y_* = the rank/position of item *Y* on the list, and *N* is the sum of the lists in the sample [58,59]. Salience characterizes the importance of an item to study participants, accounting for rank and frequency [60]. The values of the Smith’s salience scores range from 0 to 1, with values nearer to 1 depicting a higher consensus in the study population [61]. Difficulties arise in establishing and justifying salience thresholds for items on free lists [62]. Nonetheless, it has been suggested that breaks or elbows in scree plots of salience scores or frequencies of mention can be used for classifying the relevance of free-list items, when tabulated in descending order [39]. Based on elbows in the scree plots constructed from the collected data, the free list items were classified into foods of very high, high, moderate, or low salience.

For factors related to food consumption, a deductive thematic framework approach was utilized to qualitatively analyze the constructed themes from the focus group sessions. This followed a method similar to that described by Parkinson et al. (2016) [63]. The thematic framework was developed a priori, with categories centered around key areas of interest that were best-suited to the research questions. Nonetheless, some emergent sub-themes, including food-related courtship practices and parental influences on food choices, were also explored in the analyses of the data. Familiarization with the data was achieved via listening to the recorded interviews, reading the discussion transcripts, and asking the individuals who conducted the different interviews to share nuanced information about their experiences, such as the facial expressions and emotional/animated responses of participants. The responses of the participants were then summarized and organized according to the thematic categories. The data were interpreted by exploring patterns identified within them, which captured the experiences of these teenage girls relating to food consumption.

## 4. Statistical Analyses

The frequency of mention, mean ranks, and Smith’s indices for all the foods preferred and perceived as nourishing by the adolescents were extracted from the FLARES software’s output results and reported. The mean Smith’s salience indices of the top ten foods mentioned in both free lists of “preferred foods” and “foods perceived as nourishing” were compared via Student’s paired *t*-test, using R software version 4.2.2 via the RStudio environment version 2022.07.2 + 554 [64].

## 5. Results

As shown in Table 1, 48 respondents (11–16 years) from the six LGAs were interviewed. Most of the participants (46%) were between the ages of 11 and 12 years, and the mean age was 13.0 ± 2.7 years. Almost half (48%) had some primary education.

Table 2 summarizes the frequency, mean rank, and Smith’s salience indices of both the “preferred” and the “perceived as nourishing” free lists of foods reported by the adolescent girls in Bauchi State. A total of 19 foods or dishes were listed as preferred, 23 foods were considered nourishing foods, and 13 foods appeared on both the “preferred” and the “perceived to be nourishing” free lists, with varying frequencies, mean ranks, and salience scores. Pasta was the preferred food, with both the highest number of mentions (five) and the highest Smith’s index (0.62), while beans were the most frequently listed food (four times) in terms of perception as nourishing. Beans also had the highest Smith’s index (0.52). The top ten preferred foods were limited in diversity, representing only three food groups—six were starchy staples (cereals/tubers), two were legumes, and two were animal source foods. Nonetheless, the top 10 foods considered as nourishing were from four food groups—three starchy staples, one animal protein, one plant protein, and five fruits/vegetables. No fruit or dairy products, and only one vegetable, were mentioned on the complete “preferred” list. However, two fruits, four vegetables, and milk were mentioned among the foods perceived as nourishing on the complete free list. Ultra-processed foods, including instant noodles, ice cream, *Maltina* (a non-alcoholic, fermented malt drink), and *Nutrimilk* (a sweetened, flavored, packaged milk drink with a protein ratio of about 0.6 g/100 mL [65]) emerged among both the preferred foods and the foods considered nourishing. The most commonly mentioned forms of the foods included: pasta as plain pasta with oil and spices, and pasta as jollof (a one-pot dish made with pasta, chili peppers, and spices). Rice was listed as jollof as served plain with a small quantity of a side dish of tomato stew, as a one-pot dish cooked with only oil and spices, and as a one-pot porridge cooked with beans. Beans were primarily in the form of a porridge cooked only with oil and spices, or as a one-pot dish prepared with rice, pasta, or yams. Yams and potatoes were listed as fried, and served with fried egg, or fried in a plain egg batter (yam). The most commonly mentioned forms of beef were barbequed (*suya*), or spicy, shredded, and fried (*dambun nama*). Groundnuts were primarily toasted or used in thick soups. Fish was almost always fried.

**Figure 2:**
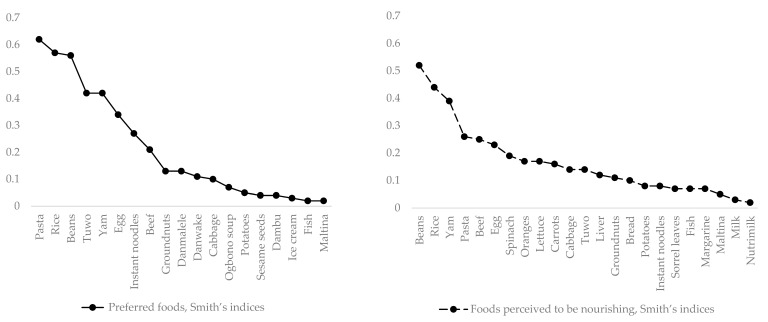
Scree plots of Smith’s salience indices of foods on the “preferred” and “perceived as nourishing” free lists of adolescent girls in Bauchi, Nigeria.

Figure 2 presents scree plots of the salience indices of the foods on the “preferred” and “perceived as nourishing” free lists generated by the study participants. On the “preferred” list, the foods occurring before the first elbow on the scree plot (pasta, rice, and beans) were classified as having very high salience; *tuwo* and yam were characterized as having high salience; egg, instant noodles, beef, and groundnuts were characterized as having moderate; and all the other foods had low salience. On the “perceived as nourishing” free list, beans, rice, and yams appeared before the first elbow and were classified as having high salience; pasta, beef, and egg were of moderate salience; and all the other foods were characterized as low in salience.

**Figure 3:**
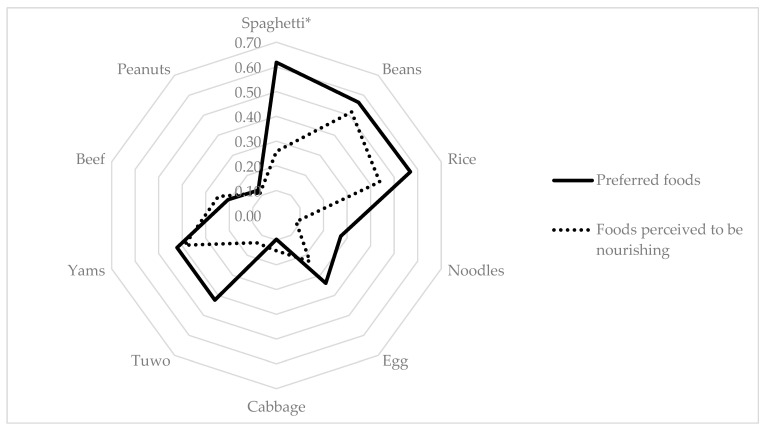
Comparison of cognitive salience according to Smith’s index of top 10 foods on both “preferred” and “perceived as nourishing” free lists of adolescent girls in Bauchi, Nigeria. * *p* < 0.05.

Figure 3 graphically depicts a comparison of the cultural salience according to Smith’s index of the top 10 foods on both the “preferred” and the “perceived as nourishing” free lists of the participants. The salience of groundnuts, beef, yam, cabbage, egg, instant noodles, rice, and beans coincided considerably (*p* > 0.05) on both free lists. However, the salience of spaghetti was much higher (*p* < 0.05) on the “preferred” than on the “perceived as nourishing” free list. *Tuwo* was higher in salience on the “preferred” free list, but the difference was not significant.

### 5.1. Study Themes

Six themes were constructed from the focus group discussions. These included knowledge about basic concepts in nutrition, food preferences, autonomy, household food allocation, marriage and courtship practices, and agricultural landscapes and economic access. Table 3 summarizes these factors.

### 5.2. Knowledge of Basic Concepts in Nutrition

Regarding their knowledge of basic concepts in nutrition, the respondents identified food items such as rice, beans, and spaghetti, and condiments such as bouillon cubes, when asked about the types of nutrients found in foods. None mentioned protein, fat, carbohydrates or any vitamin or mineral. The respondents’ responses to the types of nutrients in food are quoted below:

*“Pepper, salt and maggi* [bouillon cube]*” (Adolescent, 14 years, Darazo LGA)*

*“Rice, talea* [spaghetti]*, gwate* [porridge]*, tuwo, Indomie* [noodles]*, beans porridge, offals and meat” (Adolescent, 11 years, Darazo LGA)*

*“Maggi* [bouillon cubes]*, salt, oil, pepper and curry” (Adolescent,13 years, Ningi LGA)*

Similarly, the respondents had very limited knowledge about food groups. Some quotations on food groups and examples are as follows:

*“Milk* [fresh cow’s milk and evaporated, unsweetened]*, vegetables, pepper soup” (Adolescent, 14 years, Tafawa Balewa LGA)*

*Energy giving foods and body-building foods* *” (Adolescent, 14 years, Alkaleri LGA)*

*“Foods that make us grow fat” (Adolescent, 12 years, Alkaleri LGA)*

### 5.3. Foods That Girls and Women Should Eat

The foods that were generally considered as nourishing were presented in the free list above. The participants were questioned on which types of foods are appropriate for girls and women. The girls in three LGAs said that it was not necessary for girls, women, and children to consume any special foods, but rather to eat whatever was available in the household. Some quotations from the adolescents, in response to the question of which types of food girls and women should eat, were as follows:

*“Eggs, noodles (instant), beans, meat and macaroni” (Adolescent, 12 years, Gamawa LGA)*

*“I don’t know that there is any food specifically that girls and women should eat” (Adolescent, 12 years, Darazo LGA)*

*“Oranges, carrots, meat, fish and gwaten wake* [bean porridge]*” (Adolescent, 15 years, Darazo LGA)*

*“Any type of food available, as far as they are all food, we eat them” (Adolescent, 12 years, Shira LGA)*

### 5.4. Reasons for Food Preferences

The preferred foods are presented in the free list above. After further discussion, a small proportion of the participants reported desirable health effects, while the majority focused on the sensory qualities as reasons for their food preferences. For instance, some of the girls cited taste preferences in relation to their choice of animal source foods. Furthermore, the contribution of the food to a desirable body image for marriage was mentioned as a basis for preference. Thus, the girls liked foods that they believed would hasten weight gain, as beauty was commonly associated with not being skinny. Below are some of the responses to reasons for the food preferences on the free lists:

*“They are tasty, they promote health, they make people grow fat, they give blood” (Adolescent, 12 years, Tafawa Balewa LGA)*

*“I love ice cream because it is chilled and cools me down when I take it. After eating a meal, the sweet taste of ice cream is pleasurable” (Adolescent, 12 years, Darazo LGA)*

*“I like jollof rice and jollof spaghetti because I enjoy chewing them, unlike tuwo* [stiff cereal pudding] *that is swallowed* [without chewing]*” (Adolescent, 14 years, Darazo LGA)*

*“These foods make us look fine and we grow fat, so that when people see us, they feel like marrying us” (Adolescent, 13 years, Shira LGA)*

*“I usually like fried yam with egg, and Naman Suya* [beef barbeque]*”*[…] *We like these foods because they are very sweet* [meaning tasty]*” (Adolescent, 13 years, Ningi LGA).*

### 5.5. Autonomy

The girls’ intended age at marriage ranged from 13 to 17 years. The reasons for early marriage included a desire for independence to live and cook as they wished, since household food decisions were controlled by their parents, who often cited large family sizes and economic challenges as reasons for not providing certain foods. In the Darazo and Tafawa Balewa LGAs, a few of the girls indicated their intention to delay marriage until they were about 20 years old, so they could complete their secondary schooling.

*“I want to marry next year, so I can be independent, to cook what I want, and do everything that I want to” (Adolescent, 13 years, Darazo LGA)*

*“We are not usually given meat, milk, fish and eggs to eat at home. Our parents sometimes say the reason is because of the school fees that they have to pay, so they cannot afford these foods, since our families are large” (Adolescent, 13 years, Tafawa Balewa LGA)*

*“Me, I don’t want to marry early, because I want to go to* [secondary] *school and be educated. I don’t want to marry at less than 20 years” (Adolescent, 15 years, Darazo LGA)*

### 5.6. Gender Disparities in Household Access to Food

Inequities in household access to food were related to the quantities of the foods consumed by all the study participants, as well as the amount of animal protein foods reportedly provided by the parents to the participants in one of the six local government areas. The adolescent girls in all the LGAs reported receiving smaller food portions than the adolescent boys in their families. The perceived reasons why boys received larger food rations included their strenuous activities and contribution to agricultural labor and household income, larger body frames, and the mere fact that they were boys. Some of the girls believed that boys needed more food for strength to impregnate them. Below are some of the quotations from the respondents on food-allocation practices in the households.

*“No. Usually boys have larger portions than girls in the house. The reason is because of their nature and their body size. Another reason is that they do a lot of heavy work, so they eat a lot” (Adolescent, 13 years, Darazo LGA)*

*“The quantity given to girls and boys is not the same. Haba! Su fa maza ne* [they are boys]*!” (Adolescent, 13 years, Gamawa LGA)*

*“Boys’ and our fathers’ food at home is bigger than ours because they said they need more energy than we do. Because of the nature of their bodies and the nature of the jobs that they do. They usually go to the farm, market, and other social meetings. Also, they need strength to make babies and impregnate us” (Adolescent, 12 years, Shira LGA)*

In all the LGAs except for Alkaleri, the quantity of animal source foods reportedly given to girls was the same as that of boys.

*“Yes, both boys and girls are given the same share of animal foods” (Adolescent, 14 years, Darazo LGA)*

*“The portions of animal foods that girls receive is not equal to that of boys” (Adolescent, 11 years, Alkaleri LGA)*

### 5.7. Marriage and Courtship Practices in the Community

The prevalent age at marriage in the six LGAs ranged from 14 to 20 years. A widespread concern in the communities was that after a certain age (about 17 years), one would not be able to find a husband. However, some of the girls in Tafawa Balewa and Ningi stated their preference to complete secondary and primary schooling, respectively, before marriage. The prevalent practice of early marriage in the communities was an underlying reason for the intention of the girls to marry early and for their preference for foods that they believed would enable fast weight gain, as stated previously. Sample responses to the question of the common age at marriage in the community are summarized below:

*“At 14–17, if you have passed that age, nobody will marry you” (Adolescent, 12 years, Shira LGA)*

*“From 15 to 20 years. Nowadays, we prefer to marry when we finish secondary school after we have learned to read and write” (Adolescent, 16 years, Tafawa Balewa LGA)*

*“At 14–16 years, when we finish our* [primary] *school. If you have passed that age, people will say, you are a bad person, that is why no one wants to marry you” (Adolescent, 11 years, Ningi LGA)*

One interesting finding in this study is that prevalent courtship practices in these communities contributed to the intake of animal source foods for these girls. In response to questions related to other themes in the study, including food preferences and access to animal source proteins, the girls mentioned the role of their boyfriends/suitors in providing these foods. Despite the non-availability of these foods at home, marriage and courtship practices were related to the consumption of animal protein foods by the girls.

*“My wedding is coming up after the* [Muslim] *fasting. My husband-to-be is the one that gave me meat yesterday” (Adolescent, 14 years, Gamawa LGA)*

*“It’s only if our boyfriends come for “hira”* [evening discussion] *that they will bring suya* [beef barbeque]*” (Adolescent, 14 years, Gamawa LGA)*

*“My boyfriend usually brings suya for me, that is why I love him” (Adolescent,13 years, Ningi LGA)*

*“Usually, it’s once in a month when our boyfriends buy them* [animal source foods] *for us that we eat these foods” (Adolescent, 13 years, Shira LGA)*

### 5.8. Agricultural Landscapes and Economic Access

In Gamawa, the food preferences were limited, as only four foods were mentioned in the free lists generated by the adolescent girls. These foods were liked because they grew well in their communities. In the Gamawa, Alkaleri, Shira, and Ningi LGAs, animal protein foods including meat, milk, fish, and eggs were not usually provided at home, except on special occasions, due to the lack of affordability. The respondents’ comments on physical and economic access to foods are summarized below:

*“We like these foods* [tuwo, groundnuts, beans, sesame] *because they grow well in our community. Things like maize do not grow well, so we farm them in very small quantities” (Adolescent, 13 years, Gamawa LGA)*

*“Fish, eggs, and milk are not given* [to us] *because of lack of money” (Adolescent, 14 years, Tafawa Balewa LGA)*

*“The last time we ate meat was during the Muslim Festival* [Eid-el Kabir, popularly referred to as Big Sallah] *and since then, we have not seen meat” (Adolescent, 13 years, Alkaleri LGA)*

*“Our parents do not just kill animals for eating at home. They are usually for sale” (Adolescent, 14 years, Alkaleri LGA)*

## 6. Discussion

This study utilized the qualitative techniques of free listing and focus group discussions to explore the food preferences, perceptions of nutritive value, and factors underlying food consumption among adolescent girls in Bauchi State, Nigeria. These findings suggest that the factors that influence the diets of adolescent girls include the sensory qualities of foods, nutrition literacy, a desire for autonomy, household food allocation, courtship practices, agricultural landscapes, and economic access to food.

In Argentina, free listing was applied to a qualitative assessment of consumers’ understanding of gastronomy [58]. Additionally, this method was utilized in the comparison of parents’ and pediatricians’ perspectives on patients affected by attention deficit hyperactivity disorders [66]. In the Marshall Islands, a study generated free lists to demonstrate an improved measure of cognitive salience [67]. The salience scores of the top 10 foods ranged more broadly (0.21–0.82) in comparison with those in the present study (0.13–0.62 for preferred foods and 0.16–0.52 for foods perceived as nourishing) [67]. Nonetheless, the range of the salience indices of the complete free lists in the present study (0.02–0.62 for preferred foods and 0.02–0.52 for foods perceived as nourishing) were similar to that (0.029 to 0.613) documented in the Argentine study, which produced a free list of 28 foods representing the country’s gastronomy [58]. Perceptual salience has been shown to be a predictor of food choices [68,69]. Although previous studies focused on the impact of visual salience on food preferences, a qualitative study on adolescents’ perceptions of healthy eating in Malaysia concluded that concerns about personal health, the health conditions of others, and knowledge of healthy or unhealthy foods were among the drivers of healthy food choices [69,70]. The qualitative nature of the present study did not allow a mathematical confirmation of whether the perceptions of the nutritive value of foods were actual predictors of food preferences. However, it is important to observe the similarities in the salience levels of the top 10 foods that were preferred and perceived as nourishing in this study population.

Adequate knowledge of nutrition is crucial for the health of adolescent girls, and it influences the trajectory of maternal and future family nutrition and health [71]. The data from the present study showed inadequate knowledge concerning types of nutrients and food groups, as the participants in all the areas did not have a clear concept of nutrients, and were unable to identify any in foods. These results are similar to findings in which large proportions of adolescent girls were not able to name food groups or the food sources of nutrients [72]. Despite the higher diversity of food groups on the “perceived as nourishing” list, and the observed similarities between the top ten preferred foods and foods perceived as nourishing, gaps appear to exist in the knowledge of the study participants with respect to the nutritive value of foods. This gap is reflected by the total absence of fruits and dairy from the preferred free list, and the low salience of micronutrient-rich foods like fruits, vegetables, and dairy on the “perceived as nourishing” free list. Thus, inadequate knowledge of nutrients and food groups may be an underlying factor in the limited diversity of preferred foods, and ultimately, the food-consumption decisions and nutritional status of the adolescent girls in the current study.

Although numerous studies have shown that nutrition knowledge often does not translate into good dietary habits among adolescents, numerous recent intervention reports and reviews have shown improved dietary practices as a result of improvements in nutrition knowledge in this age group [20,73,74,75,76,77,78,79,80,81,82]. Nutrition education has been incorporated into the curricula of primary and secondary schools in Nigeria; however, recent research has shown poor levels of nutrition knowledge in public secondary schools in the country [21]. There is an urgent need to explore more effective methods for the teaching of nutrition in schools. A recent systematic review and meta-analysis has shown that school-based food and nutrition education interventions favorably influenced adolescent food consumption [80]. The incorporation of nutrition training into the *Islamiyya* non-formal educational system could be a major approach to the education of girls in this region in terms of nutrition, since approximately one-fifth of the present study’s population were part of the system, and two-thirds of the pupils in these institutions are girls [83].

Food preferences play a major role in determining dietary behaviors and food consumption [84]. One notable finding in the current study is that beans were listed as the third most preferred food by the girls, and the food that was most frequently considered nourishing. Composite dishes of cereals and legumes or pulses provide protein of improved quality due to amino acid complementarity [85,86]. In low-income countries, the use of cereal-legume mixes has been shown to be a cost-effective and feasible strategy to ameliorate protein energy undernutrition in poverty-stricken households. In these areas, animal source foods constitute only 3% of dietary calories, and 80% of dietary energy is mostly derived from cereals [85,87,88]. In the present study, the high salience of beans (the third-highest and highest on the “preferred’ and “perceived as nourishing” free lists, respectively) is a positive result, as it would make nutrition interventions advocating affordable cereal–legume mixes easily acceptable in this population [89]. Conversely, the preference of the study participants for ultra-processed foods is consistent with the evidence of the nutrition transition in rural populations [90]. In order to mitigate these dietary shifts, which are often associated with the marketing of highly processed foods to adolescents [91], the consumption of traditional foods that are nutritious, affordable, and available in the study area should be promoted [89].

A study in the United States of America showed that teenagers have little choice as to which types of food are prepared at home, as parents make decisions on household meals without seeking input from their children [92]. This is similar to the findings in the present study, in which the adolescent girls expressed their lack of autonomy in food choices, as these decisions depended on their parents. Due to this lack of autonomy, the study participants reported limited access to certain foods at home, such as animal proteins, like eggs and beef, which were of moderate salience on the free list of preferred foods. This lack of control in the present study was a motivating factor for the desire to marry early. Early marriage, often accompanied by adolescent pregnancy, has been associated with higher levels of food insecurity, undernutrition, and social, emotional, and economic consequences for girls [93,94].

The present findings add to the existing body of research on the reasons for the preference for early marriage among girls in northern Nigeria [94,95,96,97,98]. The personal interest of the girls in marrying early in this study was reflected in the young age at which they intended to marry, and coincided with the sociocultural norm of early marriage in the community. According to the Nigeria Demographic and Health Survey, the median age at marriage among women in Bauchi State is 15.5 years [11]. This figure is the lowest among the 36 states in the country. In the current research, we observed an even lower intended marital age than that indicated by the survey. The adolescent girls in this investigation identified a desire for independence from their parents, particularly with regards to food choices, as a key factor in their intention to marry early. Nonetheless, this expectation may not be realized, since married adolescent girls may often have limited autonomy and control over the decisions in their marital homes, particularly those relating to food consumption and nutrition [99,100,101,102,103]. Some authors have reported reduced risk of underweight, which is perhaps associated with increased food access through marriage [104]. However, evidence exists that early child-bearing limits linear growth in adolescent girls, since over 50% and 15% of adult weight and height, respectively, are attained between the ages of 10 and 19 years [94]. In order to redefine the norms around age at marriage in the study area, girls who decide to delay marriage to complete schooling should be supported to facilitate peer-to-peer behavior-change interventions.

Concerns regarding body image are widespread among adolescents, and they may underlie food choices [105]. The participants in the present research expressed a preference for foods believed to promote weight gain and the achievement of a body image perceived to be ideal for marriage. Body image can be an important predictor of nutritional well-being. However, there is a paucity of data on the drivers of body image perceptions, and on the relationship between marriage expectations, body image perceptions, and dietary choices in the study population [106]. Thus, these need to be explored further.

The findings from a study in Bangladesh showed that diet quality and meal frequency and quantities are often adjusted in households experiencing food insecurity [23]. In such situations, girls are often expected to make sacrifices and receive food last in the order of the household food service, to accept meals of reduced quantity and quality, and to ingest fewer foods rich in protein and micronutrients, in order to allow male family members to consume more food [23,107]. These gender dynamics often persist into marriage [23]. The present study has shown a lopsided household food distribution that favors boys over girls. This is in agreement with the findings that girls in severely food-insecure households were twice as likely to experience food insecurity than boys [108]. Although boys have higher energy requirements during adolescence, girls have elevated needs for foods with higher nutrient densities [109]. Shortfalls in the intake of energy, folate, iron, riboflavin, protein, and vitamin A have been documented in adolescent girls in Uganda [110]. Similarly, in India, adolescent girls were found to have an inadequate intake of calories, protein, calcium, and iron, in comparison to males [107]. Thus, the quality and quantities of foods consumed by adolescent girls should be increased in order to mitigate these risks.

In particular, to compensate for the increased blood losses in menstruation, fast-growing girls should consume higher quantities of foods rich in iron than boys [111]. Weekly iron and folic acid supplementation may be of benefit to adolescent girls in Bauchi State [112]. Additionally, it is established that gender stereotypes result in the socialization of girls to acknowledge their inferior social position [23]. Nonetheless, the finding in the current study that girls believed that boys needed more food to impregnate them is novel.

In Bauchi State, where over one-third of the population and 67% of farming households are food-insecure, adolescent girls are likely to experience even greater levels of food deprivation [113,114,115]. In the present research, we observed that the food preferences of the adolescent girls in the Gamawa LGA in the Bauchi North senatorial district were limited and influenced by physical access to food, particularly the types of agricultural produce thriving in their communities. The area of Gamawa has been shown to be prone to desertification, with remarkable losses of vegetation and elevation in land surface temperatures; thus, primarily drought-resistant crop varieties thrive in this arid region [116,117,118]. To improve access to healthy foods in desert-prone areas, research efforts need to be directed towards identifying and promoting the cultivation and utilization of climate-resilient, nutrient-dense foods (soybean, cowpea, and *Bambara* nuts), micronutrient-rich vegetables (Moringa leaves), and other crops that can thrive in impoverished soils [89,118].

The incidence of anemia (60.5%) is of concern for adolescent girls in Nigeria [11]. In the current research, the participants reported minimal access to animal source foods at home due to financial constraints, with many reporting access only during religious festivals. The findings from the current study concerning physical and economic access to food are similar to those reported in a study in northwestern Ethiopia. There, adolescents reported a lack of access to diverse foods due to availability issues and the restriction of access to animal source foods to periods of religious festivals [119]. Nonetheless, in our study, the girls in three LGAs reported that they only received animal source foods as gifts when their boyfriends came to visit in the evening (*hira*)*. Hira* is a voluntary, pleasurable conversation between two or more people in Hausa-speaking cultures in northern Nigeria [120]. In the case of courtship, *hira* usually involves evening visits by a suitor to his proposed bride, often accompanied with gifts [121]. The provision of animal source foods by potential husbands may be perceived as an important way to improve the protein and iron intake of the girls. Nonetheless, the failure to meet the girls’ basic food needs at home increases their susceptibility to being lured into marriage by these gifts, rather than making decisions on marriage utilizing more critical perspectives. Nutrition stakeholders should devise culturally appropriate efforts to promote home gardening and poultry production targeted at adolescent girls, in order to reduce the barriers these girls face to access to a diverse diet rich in animal source foods. Additionally, community composting of organic market wastes may improve soil fertility in desert areas [122]. Processing and preservation techniques that leverage affordable technological solutions should be applied to process and preserve the approximately 537,000 tons of milk produced annually in northern Nigeria, of which 40% is wasted [123,124,125]. One limitation of this study was that the authors did not explore the factors responsible for the differences or similarities between the top foods on both free lists. Additionally, the underlying reasons for the infrequent choices of dairy and fruits were not investigated. This is because these facts only became clear during the statistical analyses of the free lists, after the free-listing exercise and focus group discussions had been completed.

## 7. Conclusions

Optimal food consumption is crucial for promoting the health and well-being of adolescent girls, with potential intergenerational benefits. This study described the food preferences and perceptions of nutritive value and the factors influencing food consumption among adolescent girls in Bauchi State. Advocacy and programs to improve nutrition knowledge, decrease the prevalence of early marriage, increase access to animal source foods, and promote equitable household food allocation are needed to ensure the consumption of adequate food among adolescent girls in rural areas. Policy makers should focus on nutrition education and address the underlying causes of inequitable access to nutritious foods, in order to interrupt the cycle of undernutrition in these adolescent girls.

## Figures and Tables

**Table 1:** Sociodemographic characteristics of respondents (N = 48).

Characteristics	Frequency	Percent (%)
**Age group (years)**		
11–12	22	45.8
13–14	16	33.4
15–16	10	20.8
**Education**		
None	7	14.6
Islam-based	11	22.9
Some primary school	23	47.9
Some secondary school	7	14.6

**Table 2:** Characteristics of “preferred foods” and “perceived as nourishing” on free lists of adolescent girls in Bauchi, Nigeria, computed using FLARES software.

Preferred Foods	n	Mean Rank ^a^	Smith’s Salience Index ^b^	Foods Perceived to Be Nourishing	n	Mean Rank	Smith’s Salience Index
**Pasta ^c^**	5	2.60	0.62	**Beans**	4	2.25	0.52
**Rice**	4	2.25	0.57	**Rice**	3	2.00	0.44
**Beans**	5	3.20	0.56	**Yam**	3	2.33	0.39
***Tuwo ^d^***	4	4.75	0.42	**Beef**	4	5.75	0.25
**Yam**	4	4.00	0.42	**Pasta**	2	3.00	0.26
**Egg**	4	5.50	0.34	**Egg**	3	5.33	0.23
**Instant noodles**	2	3.00	0.27	Spinach	2	4.00	0.19
**Beef**	3	4.67	0.21	Oranges	1	1.00	0.17
**Groundnuts**	1	2.00	0.13	Lettuce	1	1.00	0.17
*Danmalele ^e^*	1	3.00	0.13	Carrots	2	4.50	0.16
*Danwake ^f^*	2	8.50	0.11	**Cabbage**	1	2.00	0.14
**Cabbage**	1	6.00	0.10	***Tuwo ^c^***	2	4.00	0.14
*Ogbono* soup ^g^	1	8.00	0.07	Liver	1	4.00	0.12
**Potatoes**	1	8.00	0.05	**Groundnuts**	1	3.00	0.11
Sesame seeds	1	4.00	0.04	Bread	1	5.00	0.10
*Dambu ^h^*	1	10.00	0.04	**Potatoes**	1	3.00	0.08
Ice cream	1	9.00	0.03	**Instant noodles**	1	5.00	0.08
**Fish**	1	9.00	0.02	Sorrel leaves	1	5.00	0.07
***Maltina***	1	10.00	0.02	**Fish**	1	4.00	0.07
				Margarine	1	7.00	0.07
				***Maltina ^i^***	2	8.50	0.05
				Milk	1	6.00	0.03
				*Nutrimilk ^j^*	1	10.00	0.02

^a^ Mean rank is the position in which a food item or dish appears, averaged across all food items on each free list [43]. ^b^ Smith’s salience index (*S*), which accounts for both frequency and order of mention of food items or dishes. **^c^** All foods in bold type were present on both the “preferred” and “perceived as nourishing” free lists. *^d^ Tuwo*, a stiff cereal pudding usually served with a side dish of thick soup. *^e^ Danmalele*, a sorghum pap made with palm oil and spices. *^f^ Danwake*, cooked dough balls made from cereals. ^g^ *Ogbono*, a viscous, slimy soup made from Dika nuts, popularly known as the seeds of the African mango. *^h^ Dambu*, a spicy cereal dumpling. ^i^ *Maltina*, a non-alcoholic, fermented malt drink. ^j^ *Nutrimilk*, a sweetened, flavored, packaged milk drink with protein ratio of about 0.6 g/100 mL.

**Table 3:** Factors related to food consumption of adolescent girls in rural Bauchi.

Level of Influence	Emerging Themes	Key Findings
**Intrapersonal**	Knowledge of basic concepts in nutrition	Knowledge of types of nutrients and food groups was limited Participants did not believe in any special foods for girls and women, but believed they should consume whatever foods are available at home
	Food preferences	From the free lists, food preferences were from a limited number of food groups The key factors in preferences were desirable health effects, sensory attributes, and contribution of foods to a desirable body image for marriage
	Autonomy	Foods consumed by adolescent girls were influenced by parental food choices and often limited to whatever was provided at home Desire for independence to live and cook as they wished was an incentive for early marriage Participants’ intended age at marriage ranged from 13 to 17 years
**Interpersonal**	Household food allocation	Gender inequities in household food distribution (quantity) were reported Perceived reasons for differential household food distribution included boys’ contribution to agricultural labor and the household economy, larger body frames, and the belief that boys need more food for strength to impregnate girls Household food choices depended on parents
**Sociocultural**	Marriage and courtship practices	Girls in communities married between 14 and 20 years of age, and believed that after a certain age (17 years), one would not find a husband Adolescent girls received gifts of animal protein foods from boyfriends and potential suitors as part of a community courtship practice called “*hira*” (evening discussion with a boyfriend)
**Environmental**	Agricultural landscapes and economic access	In some areas, food choices were limited because certain crops do not thrive well agriculturally Households had limited access to certain foods due to unaffordability

## Data Availability

Data are available upon reasonable request from the authors.
